# Supplementary figures and images for: An invasive appetite: Combining molecular and stable isotope analyses to reveal the diet of introduced house mice (Mus musculus) on a small, subtropical island
Source: PLoS One. 2023 Oct 19;18(10):e0293092. doi: 10.1371/journal.pone.0293092 (PMC10586637; doi:10.1371/journal.pone.0293092)

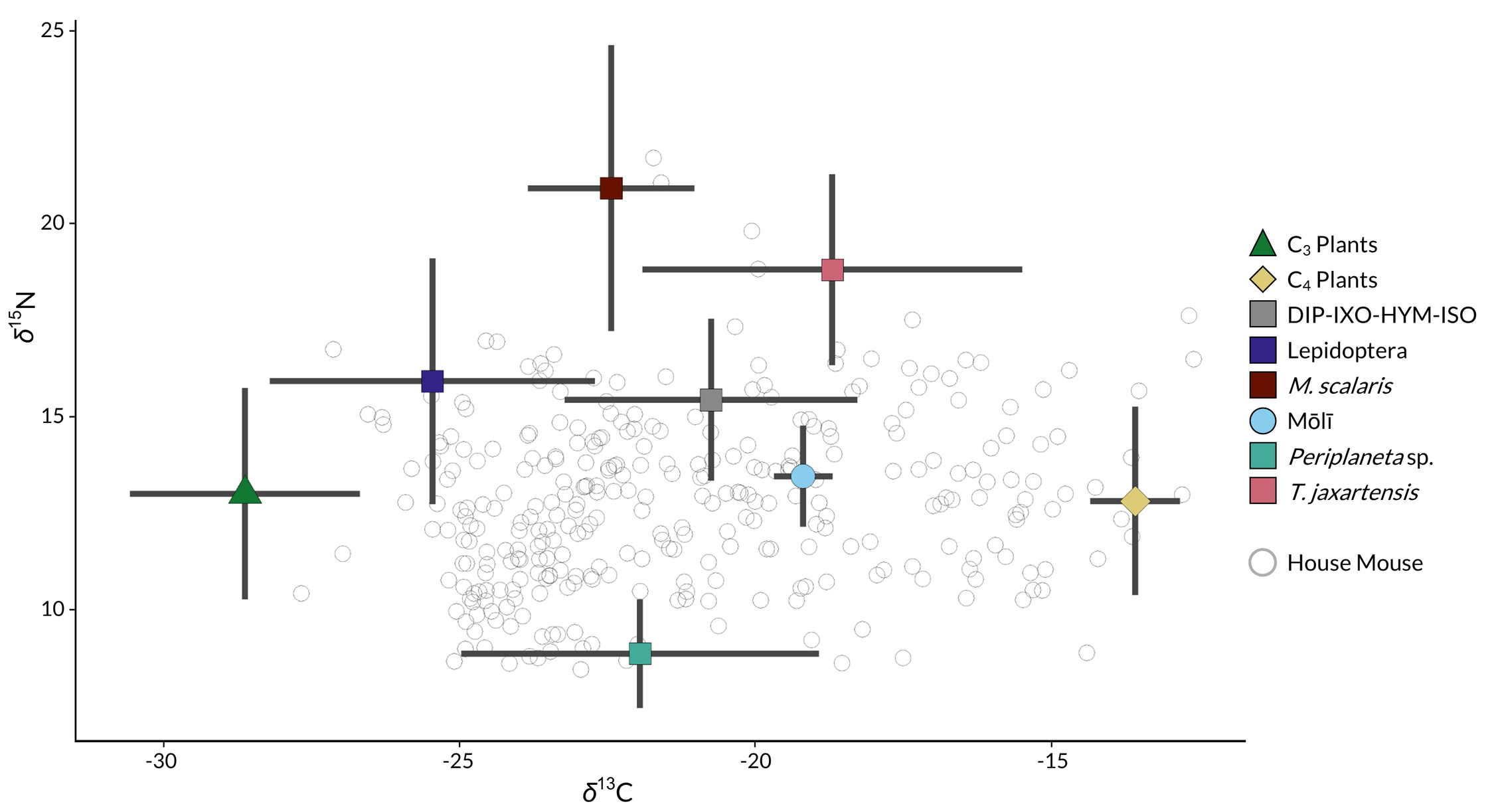

Supplement: S1 Fig — Source groups include C3 plants, C4 plants, Diptera-Ixodida-Hymenoptera-Isopoda (DIP-IXO-HYM-ISO), Lepidoptera, Megaselia scalaris, mōlī, Periplaneta sp., and Trachyzelotes jaxartensis; mean values of δ13C and δ15N indicated by source group symbol and bars represent ± 1 SD. House mouse stable isotope values were adjusted by subtracting diet-tissue discrimination factors for hair tissue (δ13C = 1.7 ± 1.3‰; δ15N = 3.2 ± 1.1‰). (TIF) [file pone.0293092.s001.tif]

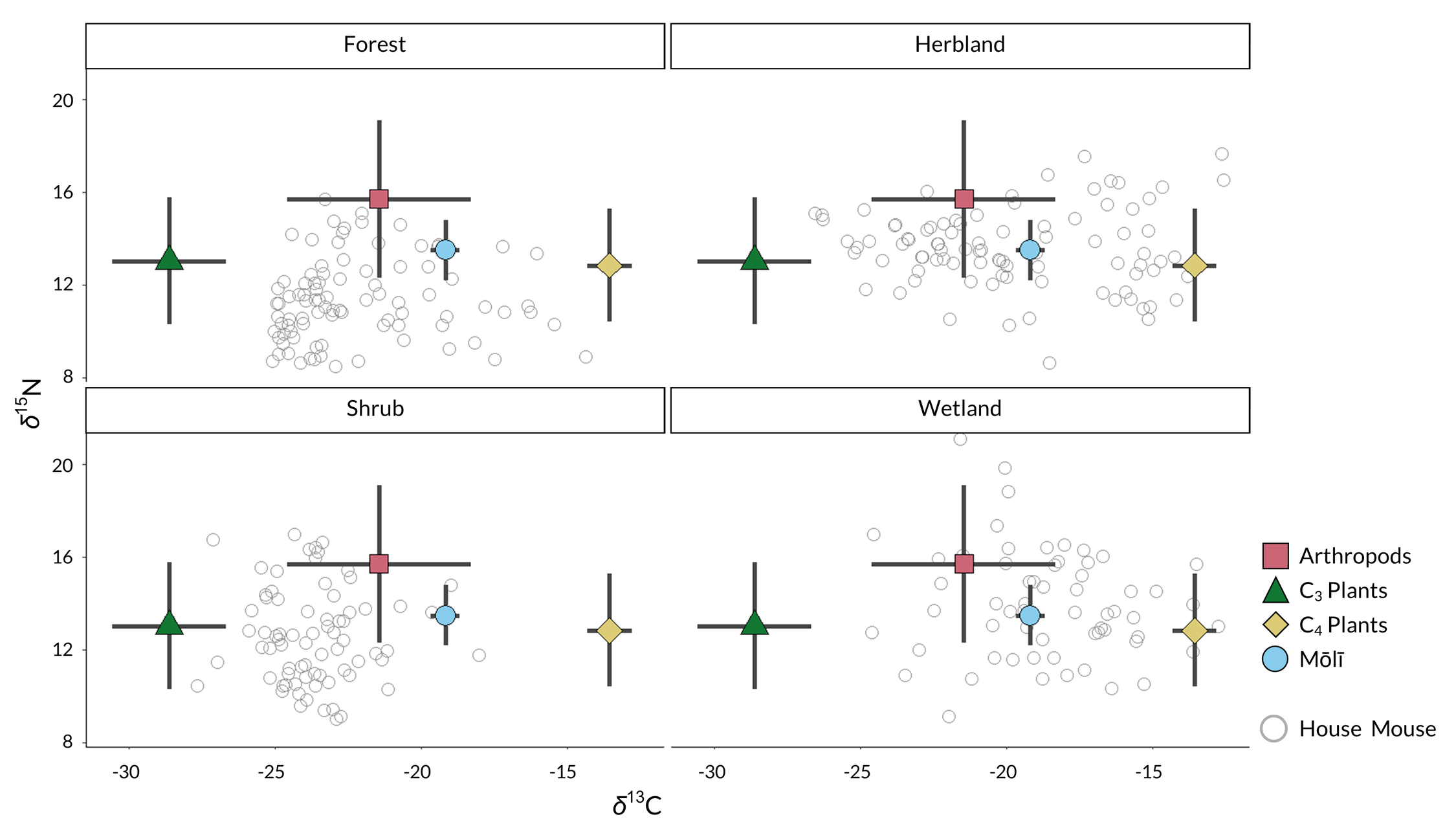

Supplement: S2 Fig — Source groups include arthropods, C3 plants, C4 plants, and mōlī; mean values of δ13C and δ15N indicated by source group symbol and bars represent ± 1 SD. House mouse stable isotope values were adjusted by subtracting diet-tissue discrimination factors for hair tissue (δ13C = 1.7 ± 1.3‰; δ15N = 3.2 ± 1.1‰). (TIF) [file pone.0293092.s002.tif]

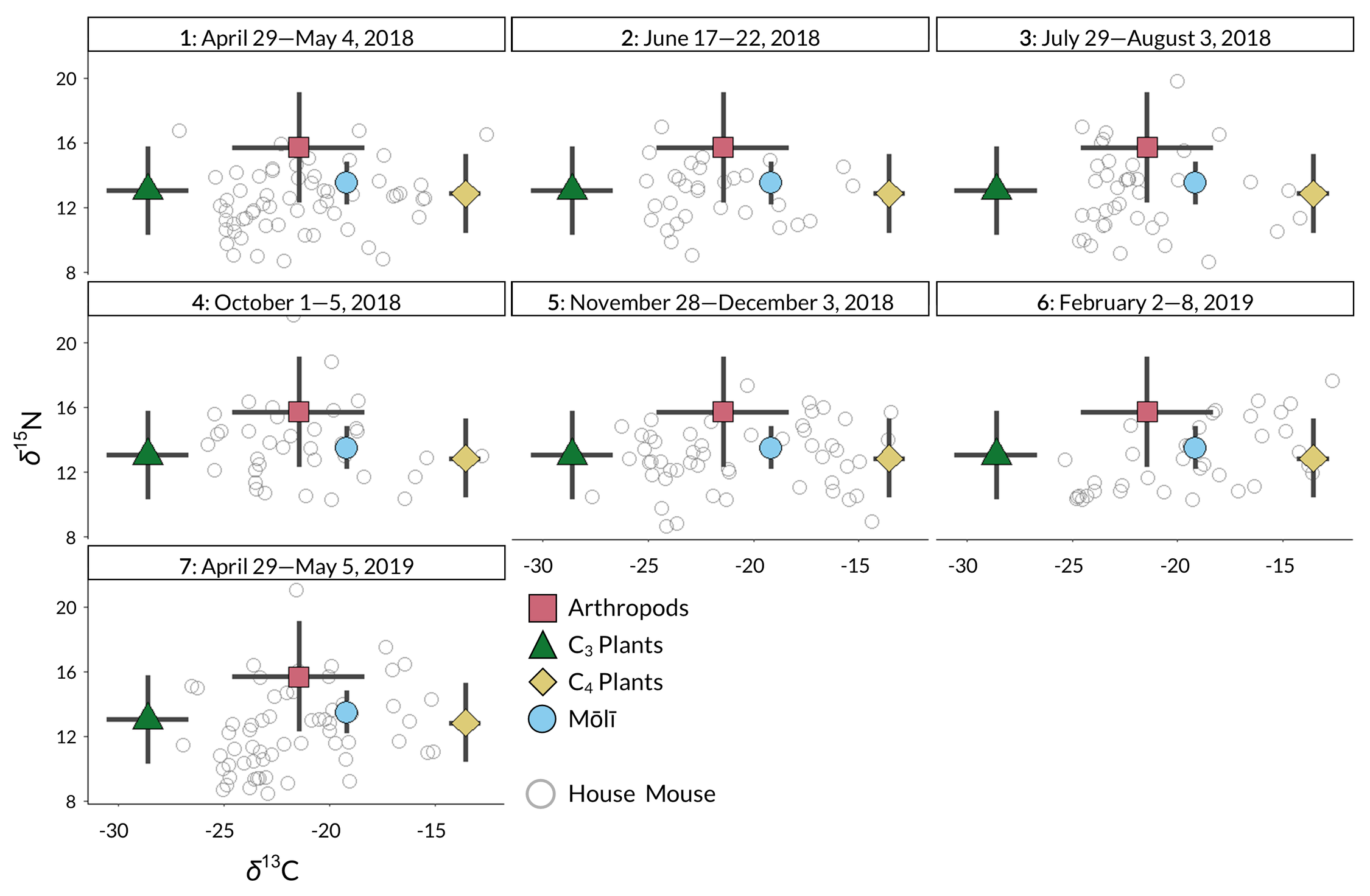

Supplement: S3 Fig — Source groups include arthropods, C3 plants, C4 plants, and mōlī; mean values of δ13C and δ15N indicated by source group symbol and bars represent ± 1 SD. House mouse stable isotope values were adjusted by subtracting diet-tissue discrimination factors for hair tissue (δ13C = 1.7 ± 1.3‰; δ15N = 3.2 ± 1.1‰). (TIF) [file pone.0293092.s003.tif]

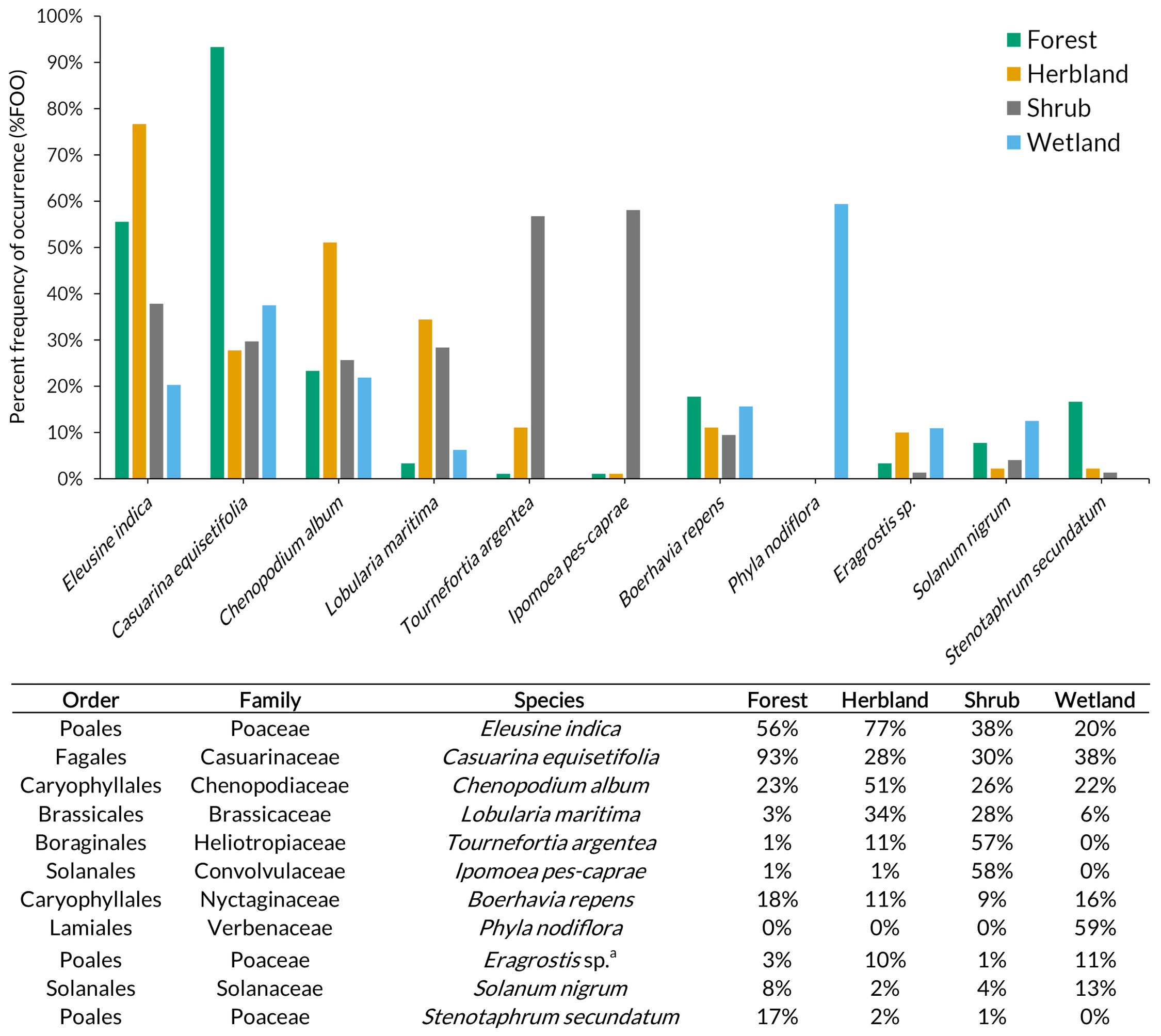

Supplement: S4 Fig — aIndicates an ASV for which native and non-native congeners exist on Kuaihelani. (TIF) [file pone.0293092.s004.tif]

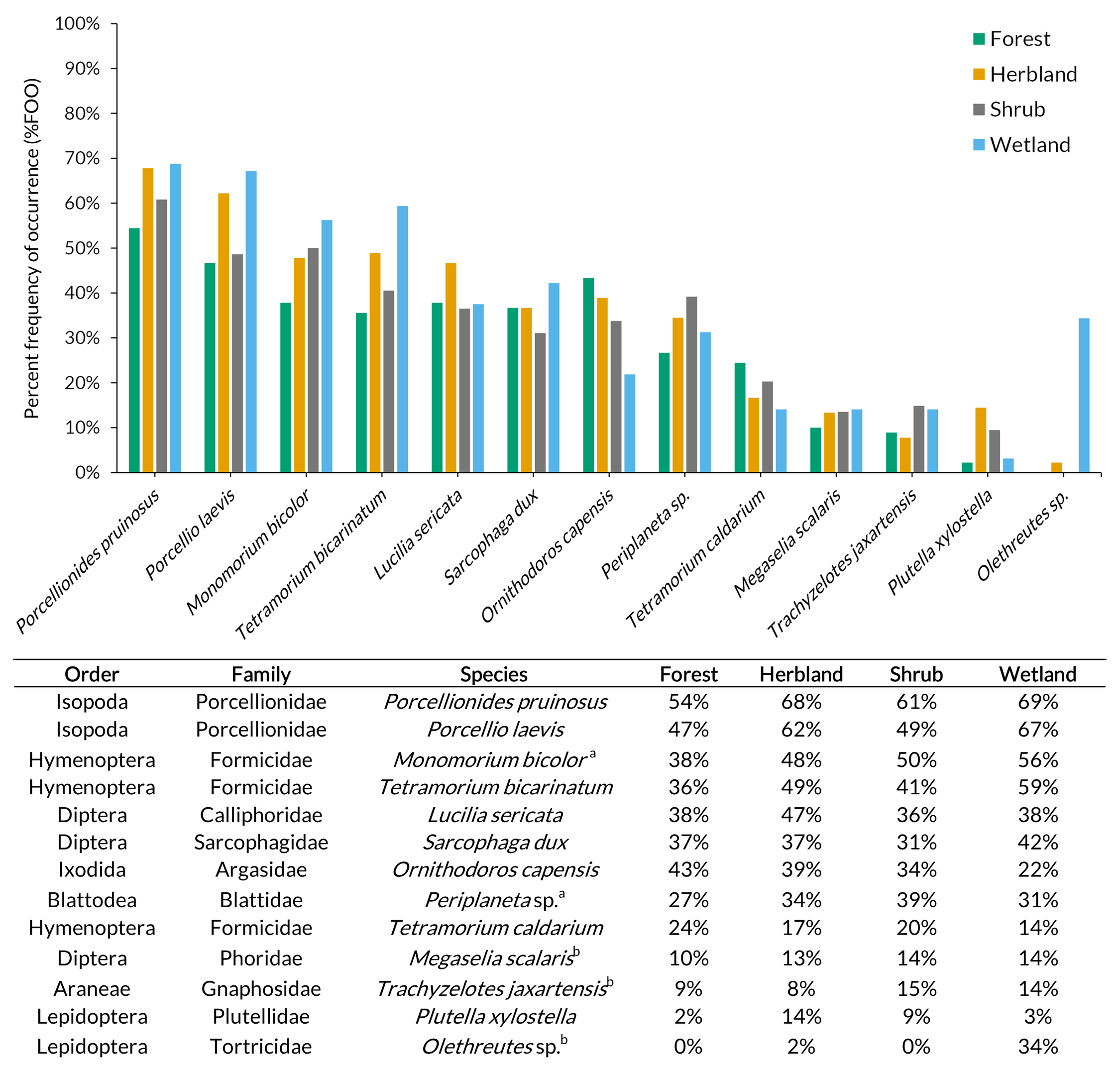

Supplement: S5 Fig — aIndicates an ASV for which native and non-native congeners exist on Kuaihelani. bIndicates an ASV that has not been observed on Kuaihelani, nor has any congeners present, and would be considered non-native. (TIF) [file pone.0293092.s005.tif]

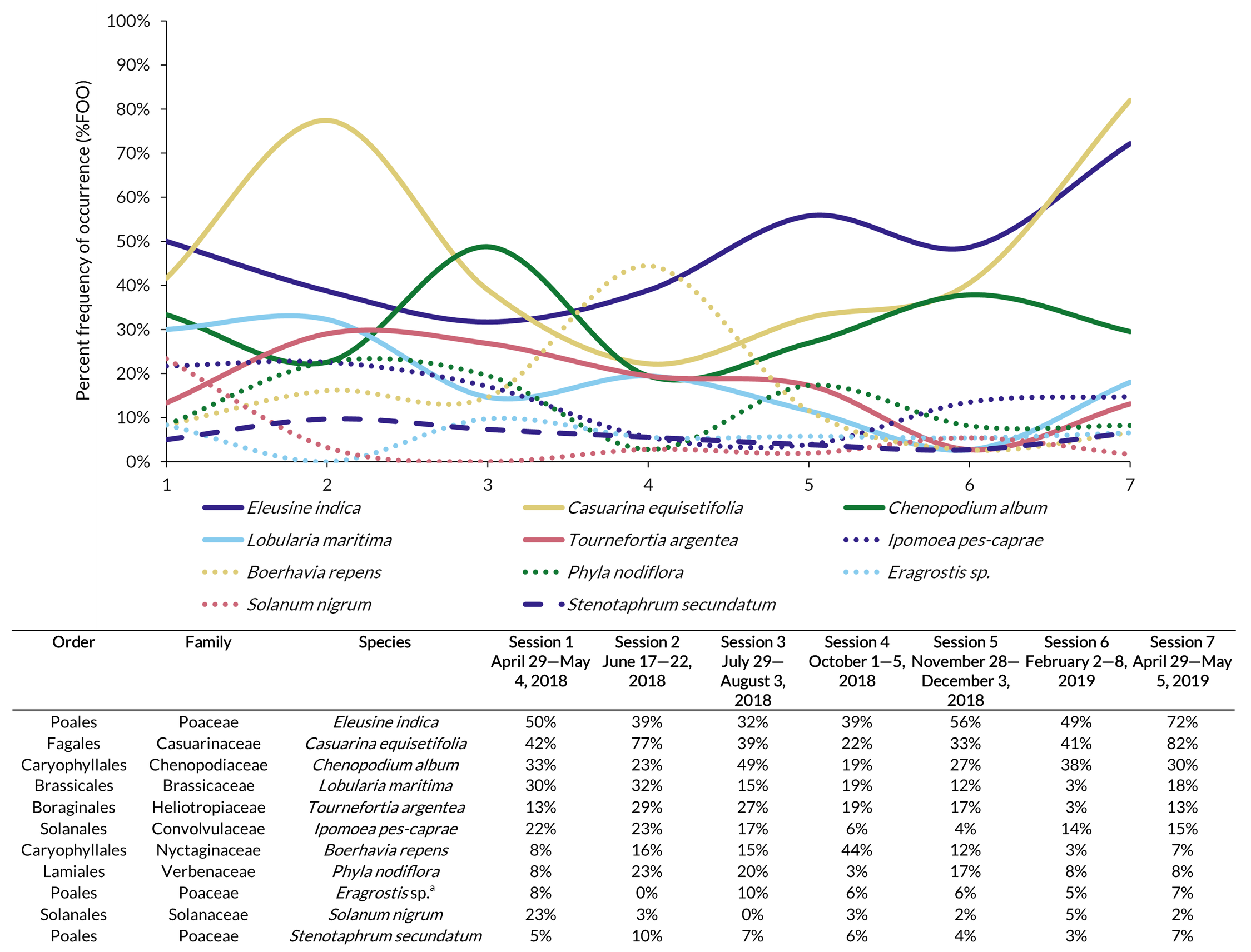

Supplement: S6 Fig — aIndicates an ASV for which native and non-native congeners exist on Kuaihelani. (TIF) [file pone.0293092.s006.tif]

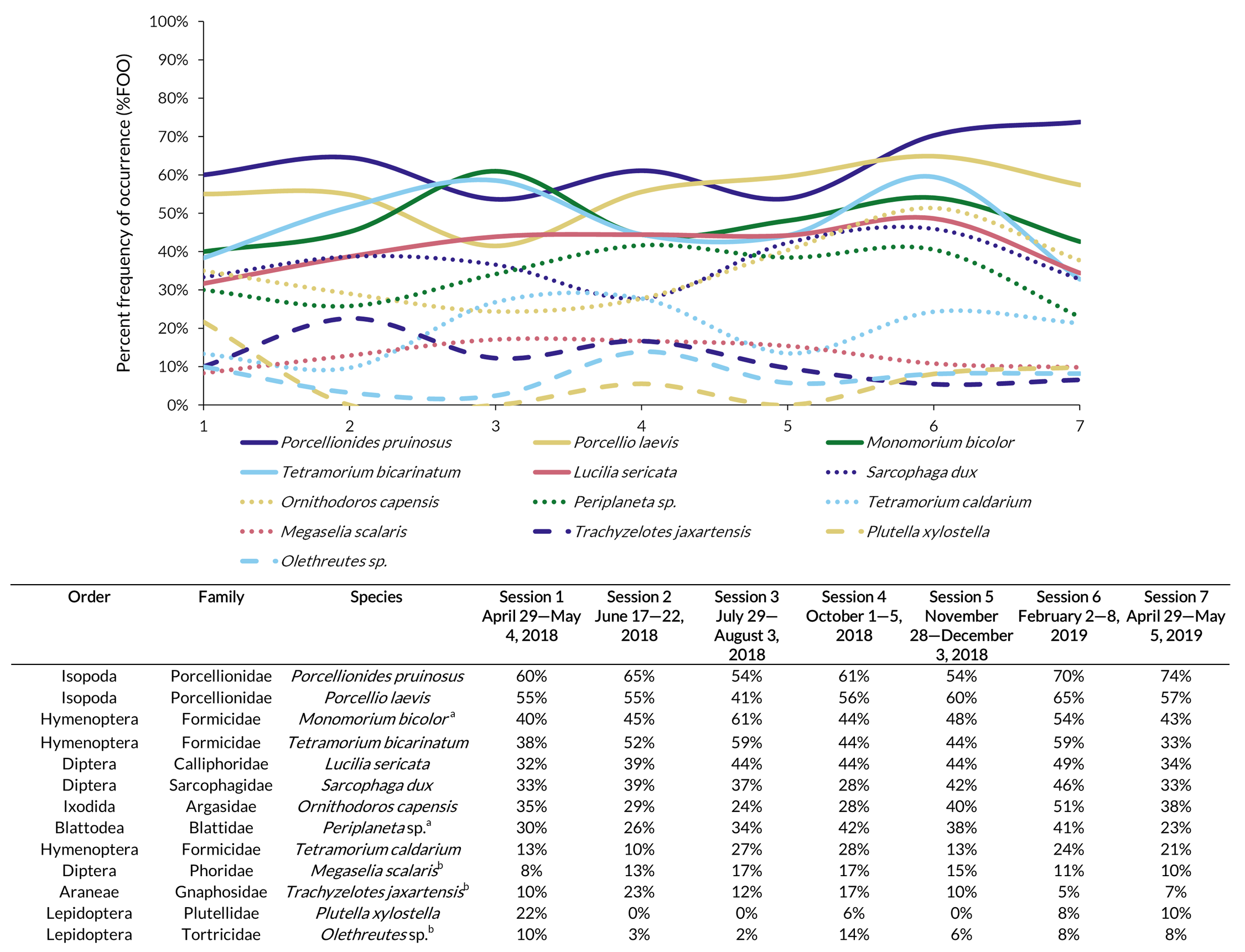

Supplement: S7 Fig — aIndicates an ASV for which native and non-native congeners exist on Kuaihelani. bIndicates an ASV that has not been observed on Kuaihelani, nor has any congeners present, and would be considered non-native. (TIF) [file pone.0293092.s007.tif]
