# Supplementary material for: An invasive appetite: Combining molecular and stable isotope analyses to reveal the diet of introduced house mice (Mus musculus) on a small, subtropical island
Source: PLoS One. 2023 Oct 19;18(10):e0293092. doi: 10.1371/journal.pone.0293092 (PMC10586637; doi:10.1371/journal.pone.0293092)
Supplement: S1 Appendix — (DOCX) [file pone.0293092.s015.docx]

Supporting Information – S1 Appendix

**An invasive appetite: combining molecular and stable isotope analyses to reveal the diet of introduced house mice (*Mus musculus*) on a small, subtropical island**

By: Wieteke A. Holthuijzen, Elizabeth N. Flint, Stefan J. Green, Jonathan H. Plissner, Daniel Simberloff, Dagmar Sweeney, Coral A. Wolf, and Holly P. Jones

*PLoS ONE*

# **S1 Appendix. Next-generation sequencing (NGS) supplemental information: PCR conditions and outcomes**

Library preparation, pooling, and sequencing were performed at the University of Illinois at Chicago (UIC) Genome Research Core in the Research Resources Center. All primer sets contained 5’ linker sequences compatible with Access Array primers for Illumina sequencers (Fluidigm, South San Francisco, USA). PCRs were performed in a total volume of 10 µL with MyTaq™ HS 2X Mix (Bioline/Meridian Bioscience, Cincinnati, USA), with primers at 500 Nm.

## **Arthropods**

Arthropod primers without CS tags used for sequencing [1,2]:

- fwhF2 5’-GGDACWGGWTGAACWGTWTAYCCHCC -3’
- fwhR2n 5’- GTRATWGCHCCDGCTARWACWGG-3’

Thermocycling conditions were as follows: 95°C for 5 min (initial denaturation), followed by 28 cycles of 95°C for 30 s, 46°C for 45 s, and 72°C for 1 min, and then one cycle of 72°C for 5 min.

For arthropods, our six DNA extraction negative controls showed minimal contamination and our six PCR-negative controls contained a mean of 0.02 ASV reads (range = 0–8 total reads) from two ASVs, neither of which was present in more than one PCR-negative control.

Prior to filtering and analysis, our 318 house mouse fecal samples generated 237,837,041 sequences, with an average of 747,915 sequences per sample. Overall, 0.84% of all sequences were assigned to Arthropoda, while 79.81% of sequences belonged to *Mus musculus*. On average, we detected 6,268 Arthropoda sequences and 593,207 *M. musculus* sequences per sample. After filtering, three samples did not contain any sequence reads. Due to the variable quality of the sequencing data, we took a conservative approach and considered an ASV present if it contributed ≥1% of the sequence reads per sample. After transforming our sequence reads to occurrence data (i.e., presence absence) and applying our 1% threshold for sequence reads per sample (for multivariate analyses), an additional 22 samples were removed (i.e., did not meet the 1% threshold).

## **Plants**

Plant primers without CS tags used for sequencing [3]:

- UniplantF 5’- TGTGAATTGCARRATYCMG -3’
- UniplantR 5’- CCCGHYTGAYYTGRGGTCDC-3’

Thermocycling conditions were as follows: 95°C for 5 min (initial denaturation), followed by 28 cycles of 95°C for 30 s, 50°C for 30 s, and 72°C for 1 min, and then one cycle of 72°C for 5 min.

For plants, our fifteen DNA extraction negative controls showed no contamination at all and our six PCR-negative controls showed a very low level of contamination. Our six PCR-negative controls contained a mean of 0.05 ASV reads (range = 0–18 total reads); the sole contaminant plant ASV was present in only one PCR-negative control. We left this plant ASV (*Eleusine indica*) in our dataset because it was present in much higher abundances across our samples (on average, ~1,017 sequence reads per sample) than in our one PCR-negative control (18 sequence reads).

Prior to filtering and analysis, our 318 house mouse fecal samples generated 7,009,993 sequences, with an average of 22,044 sequences per sample. Overall, 17.22% of all sequences were assigned to Streptophyta, and only 8.47% of sequences belonged to *Mus musculus*. On average, we detected 3,795 Streptophyta sequences and 1,867 *M. musculus* sequences per sample. After filtering, six samples did not contain any sequence reads. After transforming our sequence reads to occurrence data and applying our 1% threshold for sequence reads per sample (for multivariate analyses), no other samples were removed (i.e., all remaining samples met the 1% threshold).

## **Birds**

For birds, we experimented with two different primer sets to target cytochrome c oxidase I (COI) of the *MT-CO1* gene, neither of which was successful:

- BirdF1 5’- TTCTCCAACCACAAAGACATTGGCAC -3’ [4] and
- COIbirdR2 5’- ACGTGGGAGATAATTCCAAATCCTGG-3’ [5]
- AvMiF1* 5’- ACACTGACGACATGGTTCTACACCCCCGACATAGCATTCC -3’ [5] and
- COIbirdR2* 5’- TACGGTAGCAGAGACTTGGTCTACGTGGGAGATAATTCCAAATCCTGG-3’ [5]

**Run with PNA blocker with the following sequence: CCGTACTGCTCCTATTATCACTA*

In addition, we designed a qPCR assay to specifically amplify DNA from mōlī (Laysan Albatross – *Phoebastria immutabilis*) from house mouse fecal pellets:

- Wiet128_BirdCOI_FP* 5’- ACACTGACGACATGGTTCTACAGCYCAYGCYTTYGTAATAAT -3’
- Wiet474_BirdCOI_RP* 5’-TACGGTAGCAGAGACTTGGTCTGCNGGNGGTTTTATGTTRAT-3’

**Run with PNA blocker with the following sequence: CCGTACTGCTCCTATTATCACTA*

Thermocycling conditions were as follows: 95°C for 1 min, five cycles of 95°C for 1 min, 45°C for 40 s, and 72°C for 1 min (temperature gradient from 40-50°C), 28 cycles of 95°C for 1 min, 51°C for 40 s, 72°C for 1 min, and then one cycle of 72°C for 5 min. We also used an Asel restriction enzyme (at the restriction site ATTAAT) from ThermoFisher Scientific (Waltham, USA), according to the manufacturer’s instructions. This particular assay also did not perform successfully (like the previous two primer sets), even with the use of a peptide-nucleic acid oligonucleotide blockers and a restriction enzyme—and still only amplified house mouse DNA (as determined via sequencing).

For the qPCR assay, we first amplified genomic DNA using pre-amplification with TaqMan PreAmp Master Mix (Applied Biosystems, Waltham, USA), according to the manufacturer’s instructions because of the low target concentration. Reactions were conducted with 5 µL of genomic DNA, 10 µL of pre-amplification master mix and 5 µL of pooled TaqMan assays, and amplified using a T100 thermocycler (BioRad, Hecules, USA) with the following conditions: 10 min at 95°C, 14 cycles of 15 s at 95°C, and 4 min at 60°C. Pre-amplified samples were diluted in 1:2 ratio with TE buffer (1X Tris-EDTA Solution, Integrated DNA Technologies, Newark, USA) and used for qPCR. Primers, probes, and a double-stranded synthetic DNA standard (gBLOCKs) were synthesized by Integrated DNA Technologies (Newark, USA). All pre-amplified samples and standards were assayed using qPCR in triplicate in 10 µL reaction volumes. Each reaction contained 2.5 µL of gDNA, 5 µL of 2X TaqMan Fast Advanced Master Mix (Applied Biosystems), 0.5 µL of custom-designed 20X TaqMan Gene Expression Assay with specific minor groove binding. Ampliﬁcation and detection were performed with an ViiA7 Real Time PCR System (Applied Biosystems), under the following conditions: 2 min at 50°C to attain optimal AmpErase uracil-N-glycosylase activity, 2 min at 95°C to activate the DNA polymerase, and 40 cycles of 1 s at 95°C and 20 s at 60°C allowed for denaturation, annealing, and extension. The reporter dyes (FAM and VIC) signals were measured relative to the internal reference dye (ROX) to normalize for non-PCR-related ﬂuorescence ﬂuctuations occurring from well to well. The automatic threshold cycle (Ct) number generated after each run was used for all analyses. Again, this process still resulted in house mouse DNA amplification (as determined via sequencing).

Although we were unable to amplify bird DNA in the mouse fecal samples, there are likely several competing factors that affected our efforts. Firstly, fecal samples contain an overabundance of host (mouse) DNA in relation to target DNA, as well as gut microbiome DNA [6]. In addition, there may be challenges in using COI primers targeting very degraded bird DNA versus high quality mouse DNA, especially given that both of these organisms are eukaryotic. Despite using a variety of methods to mitigate these issues of high host DNA abundance/low target DNA abundance (including different COI primer sets, PNA blockers, restriction enzymes, and targeted qPCR assays), we still were unable to amplify bird DNA. Indeed, other studies have failed to amplify bird DNA in fecal samples (e.g., [7]); this represents an important knowledge gap that should be addressed in future studies. Field studies may provide additional difficulties in ensuring the stability of DNA, so we suggest that researchers use high-quality media (such as DNA/RNA Shield, Zymo Research, Irvine, USA) for storing and transporting nucleic acids. Additionally, DNA extraction methods can be altered to remove more host DNA [8]. For more discriminatory power—especially when host and target organisms are both eukaryotic—primer sets for different genes and genetic regions should be explored, such as 16S rRNA or Cytochrome b [9].

## **References**

1. Elbrecht V, Braukmann TWA, Ivanova NV, Prosser SWJ, Hajibabaei M, Wright M, et al. Validation of COI metabarcoding primers for terrestrial arthropods. PeerJ. 2019;10:1–23.

2. Vamos EE, Elbrecht V, Leese F. Short COI markers for freshwater macroinvertebrate metabarcoding. Metabarcoding Metagenomics. 2017;1:e14625.

3. Moorhouse-Gann RJ, Dunn JC, De Vere N, Goder M, Cole N, Hipperson H, et al. New universal ITS2 primers for high-resolution herbivory analyses using DNA metabarcoding in both tropical and temperate zones. Sci Rep. 2018;8(8542):1–15.

4. Kerr KCR, Stoeckle MY, Dove CJ, Weigt LA, Francis CM, Hebert PDN. Comprehensive DNA barcode coverage of North American birds. Mol Ecol Notes. 2007;7(4):535–43.

5. Kerr KCR, Lijtmaer DA, Barreira AS, Hebert PDN, Tubaro PL. Probing evolutionary patterns in neotropical birds through DNA barcodes. PLoS ONE. 2009;4(2):e4379.

6. Alberdi A, Aizpurua O, Bohmann K, Gopalakrishnan S, Lynggaard C, Nielsen M, et al. Promises and pitfalls of using high-throughput sequencing for diet analysis. Mol Ecol Resour. 2019;19(2):327–48.

7. Bonin M, Côté SD, Dussault C, Taillon J, Lecomte N. Combining stable isotopes, morphological, and molecular analyses to reconstruct the diet of free-ranging consumers. Ecol Evol. 2020;10:6664–76.

8. Taberlet P, Bonin A, Zinger L, Coissac E. Environmental DNA: For biodiversity research and monitoring. 1^st^ ed. Oxford, United Kingdom: Oxford University Press; 2018.

9. Andrejevic M, Markovic MK, Bursac B, Mihajlovic M, Tanasic V, Kecmanovic M, et al. Identification of a broad spectrum of mammalian and avian species using the short fragment of the mitochondrially encoded cytochrome b gene. Forensic Sci Med Pathol. 2019;15(2):169–77.
