# Supplementary material for: An invasive appetite: Combining molecular and stable isotope analyses to reveal the diet of introduced house mice (Mus musculus) on a small, subtropical island
Source: PLoS One. 2023 Oct 19;18(10):e0293092. doi: 10.1371/journal.pone.0293092 (PMC10586637; doi:10.1371/journal.pone.0293092)
Supplement: S2 Appendix — (DOCX) [file pone.0293092.s016.docx]

Supporting Information – S2 Appendix

**An invasive appetite: combining molecular and stable isotope analyses to reveal the diet of introduced house mice (*Mus musculus*) on a small, subtropical island**

By: Wieteke A. Holthuijzen, Elizabeth N. Flint, Stefan J. Green, Jonathan H. Plissner, Daniel Simberloff, Dagmar Sweeney, Coral A. Wolf, and Holly P. Jones

*PLoS ONE*

# **S2 Appendix.** **Summary of 7-source stable isotope mixing model results, excluding mōlī (Laysan Albatross, *Phoebastria immutabilis*) as a source group.**

As with the eight-source group model (including mōlī), the seven-source group model shows house mouse diet on Sand Island as being dominated by arthropods (73%), with C_3_ plants and C_4_ plants contributing nearly equal proportions (14% and 13%, respectively; S2.1 Table). Specifically, Blattodea (cockroaches; *Periplaneta* sp.) are the main arthropod prey, comprising up to 27% of mouse diet (S2.1 Table). Araneae (stealthy ground spiders; *T. jaxartensis*) and Lepidoptera (moths) both contribute 15%, while the two remaining arthropod sources (Diptera-Ixodida-Hymenoptera-Isopoda and the scuttle fly, *M. scalaris*) each constitute <10% of diet.

Arthropods are a core food source for mice across habitat types, composing 49-92% of mouse diet (S2.1 Table). Specifically, *Periplaneta* sp. are the main arthropod source in mouse diet in all habitats, except the wetland; in the herbland, in particular, the majority of mouse diet comes from *Periplaneta* sp. and *T. jaxartensis* (S2.2 Table). C_3_ and C_4_ plant contributions vary substantially among mice; C_3_ and C_4_ plants are minimally consumed in the herbland, whereas C_3_ plants are notable sources in both shrub and forest habitats (28% and 15% respectively), and C_4_ plants contribute the greatest proportion to mouse diet in wetlands (46%) (S2.2 Table).

Overall, the seven-source model results closely mirror those of the eight-source model, with minimal differences in estimated proportions by source groups both overall and among habitat types. Both models performed similarly (i.e., Gelman-Rubin statistics < 1.1 for both); when comparing estimates for each source group between models, estimates of source contributions were very similar (i.e., ≤5% difference between models). When we combined source groups *a posteriori* for each model, our eight-source model that included mōlī predicted lower arthropod contribution to house mice diet (usually by 10-15%, as compared to the seven-source model excluding mōlī), likely influenced by the fact that many detrital-feeding and necrophagous arthropods (especially the Diptera-Ixodida-Hymenoptera-Isopoda source group) and mōlī have the most similar stable isotope values of all diet items in our study.

**S2.1 Table. Summary of the estimated proportions from three (combined) source groups in house mouse diet overall and among habitat types and trapping sessions**. Source proportions are reported overall and for each habitat type and trapping session with mean values, standard deviation, and 95% credible intervals.

| **Source Group** | **Mean** | **SD** | **95% CI** |
| --- | --- | --- | --- |
| ***Overall*** |  |  |  |
| Arthropods | 73 | 13 | 43—94 |
| C_3_ Plants | 14 | 10 | 1—39 |
| C_4_ Plants | 13 | 10 | 1—39 |
| ***Forest*** |  |  |  |
| Arthropods | 80 | 14 | 51—100 |
| C_3_ Plants | 15 | 11 | 0—36 |
| C_4_ Plants | 6 | 6 | 0—19 |
| ***Herbland*** |  |  |  |
| Arthropods | 92 | 7 | 74—100 |
| C_3_ Plants | 5 | 5 | 0—17 |
| C_4_ Plants | 4 | 4 | 0—16 |
| ***Shrub*** |  |  |  |
| Arthropods | 68 | 12 | 41—90 |
| C_3_ Plants | 28 | 10 | 8—48 |
| C_4_ Plants | 4 | 4 | 0—16 |
| ***Wetland*** |  |  |  |
| Arthropods | 49 | 17 | 10—90 |
| C_3_ Plants | 5 | 8 | 0—30 |
| C_4_ Plants | 46 | 16 | 6—67 |
| ***Trapping Session 1*** |  |  |  |
| Arthropods | 72 | 14 | 40—94 |
| C_3_ Plants | 14 | 10 | 1—41 |
| C_4_ Plants | 14 | 11 | 1—41 |
| ***Trapping Session 2*** |  |  |  |
| Arthropods | 68 | 15 | 35—93 |
| C_3_ Plants | 17 | 12 | 1—47 |
| C_4_ Plants | 15 | 11 | 1—42 |
| ***Trapping Session 3*** |  |  |  |
| Arthropods | 76 | 14 | 43—96 |
| C_3_ Plants | 15 | 11 | 1—44 |
| C_4_ Plants | 10 | 8 | 1—32 |
| ***Trapping Session 4*** |  |  |  |
| Arthropods | 69 | 15 | 36—93 |
| C_3_ Plants | 17 | 12 | 2—45 |
| C_4_ Plants | 15 | 11 | 1—44 |
| ***Trapping Session 5*** |  |  |  |
| Arthropods | 72 | 14 | 41—94 |
| C_3_ Plants | 12 | 9 | 1—36 |
| C_4_ Plants | 16 | 12 | 1—46 |
| ***Trapping Session 6*** |  |  |  |
| Arthropods | 77 | 13 | 47—96 |
| C_3_ Plants | 9 | 8 | 1—29 |
| C_4_ Plants | 13 | 11 | 1—42 |
| ***Trapping Session 7*** |  |  |  |
| Arthropods | 74 | 15 | 39—95 |
| C_3_ Plants | 17 | 13 | 1—49 |
| C_4_ Plants | 9 | 8 | 1—30 |

**S2.2 Table. Summary of the estimated proportions from seven source groups in house mouse diet overall and among habitat types and trapping sessions**. Source proportions are reported overall and for each habitat type and trapping session with mean values, standard deviation, and 95% credible intervals.

| **Source Group** | **Mean** | **SD** | **95% CI** |
| --- | --- | --- | --- |
| ***Overall*** |  |  |  |
| C_3_ Plants | 14 | 10 | 1—39 |
| C_4_ Plants | 13 | 10 | 1—39 |
| Diptera-Ixodida-Hymenoptera-Isopoda | 8 | 8 | 0—29 |
| Lepidoptera | 15 | 11 | 1—41 |
| *Megaselia scalaris* | 7 | 8 | 0—28 |
| *Periplaneta* sp. | 27 | 13 | 6—55 |
| *Trachyzelotes jaxartensis* | 15 | 10 | 2—40 |
| ***Forest*** |  |  |  |
| C_3_ Plants | 15 | 11 | 0—36 |
| C_4_ Plants | 6 | 6 | 0—19 |
| Diptera-Ixodida-Hymenoptera-Isopoda | 7 | 7 | 0—26 |
| Lepidoptera | 9 | 8 | 0—27 |
| *Megaselia scalaris* | 4 | 5 | 0—17 |
| *Periplaneta* sp. | 53 | 10 | 35—73 |
| *Trachyzelotes jaxartensis* | 6 | 5 | 0—17 |
| ***Herbland*** |  |  |  |
| C_3_ Plants | 5 | 5 | 0—17 |
| C_4_ Plants | 4 | 4 | 0—16 |
| Diptera-Ixodida-Hymenoptera-Isopoda | 4 | 5 | 0—19 |
| Lepidoptera | 5 | 5 | 0—18 |
| *Megaselia scalaris* | 2 | 3 | 0—9 |
| *Periplaneta* sp. | 41 | 8 | 21—57 |
| *Trachyzelotes jaxartensis* | 41 | 8 | 26—56 |
| ***Shrub*** |  |  |  |
| C_3_ Plants | 28 | 10 | 8—48 |
| C_4_ Plants | 4 | 4 | 0—16 |
| Diptera-Ixodida-Hymenoptera-Isopoda | 8 | 8 | 0—28 |
| Lepidoptera | 16 | 11 | 0—38 |
| *Megaselia scalaris* | 8 | 8 | 0—28 |
| *Periplaneta* sp. | 33 | 9 | 18—50 |
| *Trachyzelotes jaxartensis* | 3 | 3 | 0—11 |
| ***Wetland*** |  |  |  |
| C_3_ Plants | 5 | 8 | 0—30 |
| C_4_ Plants | 46 | 15 | 6—67 |
| Diptera-Ixodida-Hymenoptera-Isopoda | 2 | 4 | 0—9 |
| Lepidoptera | 24 | 15 | 0—47 |
| *Megaselia scalaris* | 2 | 5 | 0—22 |
| *Periplaneta* sp. | 8 | 9 | 0—33 |
| *Trachyzelotes jaxartensis* | 13 | 14 | 0—46 |
| ***Trapping Session 1*** |  |  |  |
| C_3_ Plants | 14 | 10 | 2—41 |
| C_4_ Plants | 14 | 11 | 1—41 |
| Diptera-Ixodida-Hymenoptera-Isopoda | 8 | 8 | 0—29 |
| Lepidoptera | 13 | 10 | 1—40 |
| *Megaselia scalaris* | 6 | 7 | 0—27 |
| *Periplaneta* sp. | 30 | 14 | 6—59 |
| *Trachyzelotes jaxartensis* | 14 | 10 | 2—39 |
| ***Trapping Session 2*** |  |  |  |
| C_3_ Plants | 17 | 12 | 1—47 |
| C_4_ Plants | 15 | 11 | 1—42 |
| Diptera-Ixodida-Hymenoptera-Isopoda | 9 | 10 | 0—34 |
| Lepidoptera | 17 | 12 | 1—47 |
| *Megaselia scalaris* | 8 | 9 | 0—35 |
| *Periplaneta* sp. | 23 | 12 | 4—50 |
| *Trachyzelotes jaxartensis* | 12 | 8 | 1—32 |
| ***Trapping Session 3*** |  |  |  |
| C_3_ Plants | 15 | 11 | 1—44 |
| C_4_ Plants | 10 | 8 | 1—32 |
| Diptera-Ixodida-Hymenoptera-Isopoda | 8 | 9 | 0—32 |
| Lepidoptera | 20 | 14 | 1—54 |
| *Megaselia scalaris* | 8 | 10 | 0—35 |
| *Periplaneta* sp. | 26 | 13 | 5—54 |
| *Trachyzelotes jaxartensis* | 13 | 9 | 1—37 |
| ***Trapping Session 4*** |  |  |  |
| C_3_ Plants | 17 | 12 | 2—45 |
| C_4_ Plants | 15 | 11 | 1—44 |
| Diptera-Ixodida-Hymenoptera-Isopoda | 9 | 10 | 0—35 |
| Lepidoptera | 18 | 13 | 1—48 |
| *Megaselia scalaris* | 9 | 9 | 0—35 |
| *Periplaneta* sp. | 20 | 11 | 4—45 |
| *Trachyzelotes jaxartensis* | 13 | 10 | 1—38 |
| ***Trapping Session 5*** |  |  |  |
| C_3_ Plants | 12 | 9 | 1—36 |
| C_4_ Plants | 16 | 12 | 1—46 |
| Diptera-Ixodida-Hymenoptera-Isopoda | 7 | 8 | 0—29 |
| Lepidoptera | 11 | 9 | 1—33 |
| *Megaselia scalaris* | 7 | 7 | 0—28 |
| *Periplaneta* sp. | 32 | 14 | 7—61 |
| *Trachyzelotes jaxartensis* | 15 | 11 | 1—42 |
| ***Trapping Session 6*** |  |  |  |
| C_3_ Plants | 9 | 8 | 1—29 |
| C_4_ Plants | 13 | 11 | 1—42 |
| Diptera-Ixodida-Hymenoptera-Isopoda | 7 | 9 | 0—29 |
| Lepidoptera | 11 | 9 | 1—34 |
| *Megaselia scalaris* | 5 | 6 | 0—22 |
| *Periplaneta* sp. | 29 | 14 | 6—59 |
| *Trachyzelotes jaxartensis* | 24 | 15 | 3—59 |
| ***Trapping Session 7*** |  |  |  |
| C_3_ Plants | 17 | 13 | 1—47 |
| C_4_ Plants | 9 | 8 | 1—30 |
| Diptera-Ixodida-Hymenoptera-Isopoda | 7 | 7 | 0—27 |
| Lepidoptera | 17 | 13 | 1—49 |
| *Megaselia scalaris* | 6 | 8 | 0—29 |
| *Periplaneta* sp. | 29 | 14 | 6—58 |
| *Trachyzelotes jaxartensis* | 16 | 10 | 2—41 |
